# Supplementary material for: Ventral tegmental area dopamine projections to the hippocampus trigger long-term potentiation and contextual learning
Source: Nat Commun. 2024 May 21;15:4100. doi: 10.1038/s41467-024-47481-4 (PMC11109191; doi:10.1038/s41467-024-47481-4)
Supplement: Supplementary file 2 — Reporting Summary [file 41467_2024_47481_MOESM2_ESM.pdf]

nature portfolio

Corresponding author(s): Lionel Dahan

Last updated by author(s): Feb 25, 2024

Reporting Summary

Nature Portfolio wishes to improve the reproducibility of the work that we publish. This form provides structure for consistency and transparency in reporting. For further information on Nature Portfolio policies, see our Editorial Policies and the Editorial Policy Checklist.

Statistics

For all statistical analyses, confirm that the following items are present in the figure legend, table legend, main text, or Methods section.

n/a	Confirmed	
☐	☒	The exact sample size (*n*) for each experimental group/condition, given as a discrete number and unit of measurement
☐	☒	A statement on whether measurements were taken from distinct samples or whether the same sample was measured repeatedly
☐	☒	The statistical test(s) used AND whether they are one- or two-sided<br>*Only common tests should be described solely by name; describe more complex techniques in the Methods section.*
☒	☐	A description of all covariates tested
☐	☒	A description of any assumptions or corrections, such as tests of normality and adjustment for multiple comparisons
☐	☒	A full description of the statistical parameters including central tendency (e.g. means) or other basic estimates (e.g. regression coefficient) AND variation (e.g. standard deviation) or associated estimates of uncertainty (e.g. confidence intervals)
☐	☒	For null hypothesis testing, the test statistic (e.g. *F*, *t*, *r*) with confidence intervals, effect sizes, degrees of freedom and *P* value noted<br>*Give P values as exact values whenever suitable.*
☒	☐	For Bayesian analysis, information on the choice of priors and Markov chain Monte Carlo settings
☒	☐	For hierarchical and complex designs, identification of the appropriate level for tests and full reporting of outcomes
☒	☐	Estimates of effect sizes (e.g. Cohen's *d*, Pearson's *r*), indicating how they were calculated

Our web collection on statistics for biologists contains articles on many of the points above.

Software and code

Policy information about availability of computer code

Data collection	Electrophysiology data were collected using Spike2 v7.0 (Cambridge Electronic Design).
Data analysis	Electrophysiology data were analyzed using Spike2 v7.0 (Cambridge Electronic Design). Confocal images were generated using LasX 1.4.5 (Leica microsystems) and analyzed with ImageJ (version 1.53t). Power calculations were done using Minitab v18 and statistical analysis were done using GraphPad Prism 8.

For manuscripts utilizing custom algorithms or software that are central to the research but not yet described in published literature, software must be made available to editors and reviewers. We strongly encourage code deposition in a community repository (e.g. GitHub). See the Nature Portfolio guidelines for submitting code & software for further information.

Data

Policy information about availability of data

All manuscripts must include a data availability statement. This statement should provide the following information, where applicable:

- Accession codes, unique identifiers, or web links for publicly available datasets
- A description of any restrictions on data availability
- For clinical datasets or third party data, please ensure that the statement adheres to our policy

Data are available in the Source Data file provided with the article. All electrophysiological and video raw data are available upon request, as stated in the data availability statement in the manuscript.

Human research participants

Policy information about studies involving human research participants and Sex and Gender in Research.

Reporting on sex and gender	N/A
Population characteristics	N/A
Recruitment	N/A
Ethics oversight	N/A

Note that full information on the approval of the study protocol must also be provided in the manuscript.

Field-specific reporting

Please select the one below that is the best fit for your research. If you are not sure, read the appropriate sections before making your selection.

☒ Life sciences ☐ Behavioural & social sciences ☐ Ecological, evolutionary & environmental sciences

For a reference copy of the document with all sections, see nature.com/documents/nr-reporting-summary-flat.pdf

Life sciences study design

All studies must disclose on these points even when the disclosure is negative.

Sample size	Number of animals per group was calculated using minitab and aiming at 90% statistical power.
Data exclusions	Mice showing >33.33% freezing in the alternative context, thus exhibiting generalization, were excluded from the analysis (1 from non-pre-exposed mice and 4 YFP).
Replication	The main finding of this study presented in fig 2a (concomittant activation of schaffer collatrals and dopamine terminals trigger LTP) is replicated in fig 2b to 2f. All these replications were succesful and no other attempt has been tried. No attempt of replication has been conducted for other experiments presented in this study.
Randomization	Randomization was made by tossing a coin and each batch contained balanced sample size of each experimental group.
Blinding	For electrophysiology, blinding was not possible because the same experiementer was doing the experiment and analyzing the data online. For all other experiments, the experimenter was blind to the experimental group when performing experiments and analysing the data.

Reporting for specific materials, systems and methods

We require information from authors about some types of materials, experimental systems and methods used in many studies. Here, indicate whether each material, system or method listed is relevant to your study. If you are not sure if a list item applies to your research, read the appropriate section before selecting a response.

Materials & experimental systems

n/a	Involved in the study	
☐	☒	Antibodies
☒	☐	Eukaryotic cell lines
☒	☐	Palaeontology and archaeology
☐	☒	Animals and other organisms
☒	☐	Clinical data
☒	☐	Dual use research of concern

Methods

n/a	Involved in the study	
☒	☐	ChIP-seq
☒	☐	Flow cytometry
☒	☐	MRI-based neuroimaging

Antibodies

Antibodies used	Primary antibodies : goat anti-YFP 1:2500 (Rockland, 600101215) and rabbit anti-TH 1:1000 (Millipore, AB152). Secondary antibodies : donkey anti-goat A488 1:250 (Thermofisher, A11055) and donkey anti-rabbit A555 1:250 (Thermofisher, A31572).

Validation	The GFP and TH primary antibodies have been validated for Immunofluorescence by the manufacturer and used in several publications referenced on the manufacturer's website at the following address : https://www.rockland.com/categories/primary-antibodies/gfp-antibody-600-101-215/ https://www.merckmillipore.com/FR/fr/product/Anti-Tyrosine-Hydroxylase-Antibody,MM_NF-AB152

Animals and other research organisms

Policy information about studies involving animals; ARRIVE guidelines recommended for reporting animal research, and Sex and Gender in Research

Laboratory animals	mice, DAT::Cre (backcrossed on C57Bl/6J), 2 to 6,5 months old
Wild animals	study did not involve wild animals
Reporting on sex	At the outset of this project, several years ago, our primary objective was to minimize the sample size for ethical considerations. The existing literature indicated that estrogen variation could potentially lead to heightened variability in long-term potentiation (LTP) and freezing behavior among females. As a result, the study initially included only males. Over time, we have come to appreciate the importance of examining both males and females from an equality standpoint. Ongoing research now systematically incorporates mice of both sexes, actively addressing this aspect and underscoring our commitment to a more thorough and impartial investigation.
Field-collected samples	study did not involve samples collected from the field
Ethics oversight	All experiments were performed in accordance with the recommendations of the European Union (86/609/EEC) and the french National Committee (87/848) under the guidance of the local ethical committee for animal experimentation of the Federation de Recherche en Biologie de Toulouse (FRBT).

Note that full information on the approval of the study protocol must also be provided in the manuscript.
